# Dopaminergic modulation of synaptic transmission and neuronal activity patterns in the zebrafish homolog of olfactory cortex

**DOI:** 10.3389/fncir.2012.00076

**Published:** 2012-10-23

**Authors:** Yan-Ping Zhang Schärer, Jennifer Shum, Anastasios Moressis, Rainer W. Friedrich

**Affiliations:** ^1^Friedrich Miescher Institute for Biomedical ResearchBasel, Switzerland; ^2^University of BaselBasel, Switzerland

**Keywords:** dopamine, zebrafish, olfactory cortex, telencephalon, synaptic modulation, activity pattern

## Abstract

Dopamine (DA) is an important modulator of synaptic transmission and plasticity that is causally involved in fundamental brain functions and dysfunctions. We examined the dopaminergic modulation of synaptic transmission and sensory responses in telencephalic area Dp of zebrafish, the homolog of olfactory cortex. By combining anatomical tracing and immunohistochemistry, we detected no DA neurons in Dp itself but long-range dopaminergic input from multiple other brain areas. Whole-cell recordings revealed no obvious effects of DA on membrane potential or input resistance in the majority of Dp neurons. Electrical stimulation of the olfactory tracts produced a complex sequence of synaptic currents in Dp neurons. DA selectively decreased inhibitory currents with little or no effect on excitatory components. Multiphoton calcium imaging showed that population responses of Dp neurons to olfactory tract stimulation or odor application were enhanced by DA, consistent with its effect on inhibitory synaptic transmission. These effects of DA were blocked by an antagonist of D2-like receptors. DA therefore disinhibits and reorganizes sensory responses in Dp. This modulation may affect sensory perception and could be involved in the experience-dependent modification of odor representations.

## Introduction

Olfactory cortex is an evolutionarily old, paleocortical brain area that processes odor-evoked activity patterns transmitted from the olfactory bulb (OB). In mammals, olfactory cortex is further subdivided into multiple areas including anterior olfactory, piriform, and lateral entorhinal cortex. Principal neurons in olfactory cortex receive excitatory input from mitral cells associated with different glomeruli (Miyamichi et al., 2011) and from distributed subsets of other cortical neurons (Johnson et al., 2000; Neville and Haberly, 2004; Franks et al., 2011). In addition, multiple types of local GABAergic interneurons mediate strong feed-forward and feed-back inhibition (Neville and Haberly, 2004; Suzuki and Bekkers, 2007; Stokes and Isaacson, 2010). The distributed connectivity between the OB and olfactory cortex, as well as within cortex, implies that responses of cortical neurons are determined by activity patterns across multiple processing channels in the OB. Responses of cortical neurons to odor mixtures cannot easily be predicted from responses to the individual components, and response profiles to odors are shaped by both excitation and inhibition (Yoshida and Mori, 2007; Barnes et al., 2008; Stettler and Axel, 2009; Yaksi et al., 2009). Olfactory cortex therefore establishes representations of odor objects by combining information about molecular features (Wilson and Sullivan, 2011).

In addition, olfactory cortex is likely to have other functions. In the zebrafish homolog of olfactory cortex, temporal filtering tunes neurons to those features of OB output patterns that are particularly informative about precise odor identity (Blumhagen et al., 2011). In piriform cortex of anesthetized rats, the intensity of odor responses is modulated together with the global brain state, suggesting that olfactory cortex acts as a state-dependent sensory gate (Murakami et al., 2005). Modeling studies and recent experimental evidence suggest that olfactory cortex stores information about specific odors by modifying pattern completion and pattern separation. This process is assumed to depend on a global teaching signal that enables synaptic plasticity when a pattern is being stored but disables plasticity during recall (Hasselmo et al., 1990; Hasselmo, 1993; Barnes et al., 2008; Chapuis and Wilson, 2011; Wilson and Sullivan, 2011).

The function of cortical circuits is influenced by neuromodulators. In olfactory cortex, stimulation of muscarinic acetylcholine receptors decreased synaptic inhibition of pyramidal cells in superficial layers and facilitated the induction of long-term potentiation at cortico-cortical excitatory synapses, possibly as a consequence of dendritic disinhibition (Kanter and Haberly, 1993; Hasselmo and Barkai, 1995; Patil et al., 1998; Patil and Hasselmo, 1999). It has therefore been proposed that cholinergic input, which is thought to convey information about salient events, acts as a contextual signal that enables synaptic plasticity when odor-evoked activity patterns are stored. Another neuromodulator that conveys information about important environmental events is dopamine (DA). In rodents, olfactory cortical neurons receive dopaminergic input from midbrain nuclei, particularly the ventral tegmental area (Harvey et al., 1975; Fallon and Moore, 1978; Datiche and Cattarelli, 1996). Midbrain DA neurons broadcast reward-related signals, but also other signals including information about unexpected events, to a wide range of targets in the forebrain (Waelti et al., 2001; Schultz, 2002; Redgrave and Gurney, 2006). In many of these areas, DA modulates synaptic transmission and plasticity (Otmakhova and Lisman, 1996, 1998; Gurden et al., 2000; Schultz, 2002; Pawlak et al., 2010), but few studies examined dopaminergic effects in olfactory cortex (Legge et al., 1966; Collins et al., 1985; Gellman and Aghajanian, 1993). The influence of the dopaminergic system on cortical odor processing is therefore unclear.

We examined effects of DA in a higher olfactory brain area of zebrafish. As in other species, odor stimulation in zebrafish activates distributed combinations of glomeruli in the OB (Friedrich and Korsching, 1997). Processing of glomerular activity patterns by neuronal circuits within the zebrafish OB results in decorrelated activity patterns across the output neurons, the mitral cells (Friedrich and Laurent, 2001; Friedrich et al., 2004). These activity patterns are stable against small variations in the stimulus but change abruptly when the stimulus is modified more substantially, indicating that the OB establishes locally stable, discrete and combinatorial representations of odors (Niessing and Friedrich, 2010). Output from the OB is conveyed to multiple higher brain areas including the posterior zone of the dorsal telencephalon (Dp) of zebrafish, the homolog of mammalian olfactory cortex that may correspond specifically to piriform cortex (Northcutt, 1981; Wullimann and Mueller, 2004; Mueller et al., 2011). Unlike mitral cells, Dp neurons often respond to a binary mixture of odors in a mixture-specific fashion, suggesting that Dp is involved in the representation of olfactory objects (Yaksi et al., 2009). Furthermore, read-out of mitral cell activity patterns by neuronal circuits in Dp results in distinct representations of similar odors (Blumhagen et al., 2011) which is thought to facilitate the storage of information by auto-associative networks performing pattern separation and completion (Wilson and Sullivan, 2011). However, the role of Dp in olfactory learning and memory has not yet been directly examined.

In this study, we examined effects of DA on synaptic transmission and odor-evoked activity patterns in Dp. Neurons in Dp and other pallial brain areas are not arranged in distinct layers because the development of the teleost pallium does not follow the inside-out pattern of cortical development in other vertebrate classes (Northcutt, 1981; Wullimann and Mueller, 2004; Mueller et al., 2011). Nevertheless, Dp exhibits obvious functional similarities to mammalian piriform cortex. For example, odors evoke distributed and odor-specific activity patterns that involve the convergence of processing channels in the OB, represent odor-objects, and are strongly shaped by inhibition (Yaksi et al., 2009; Blumhagen et al., 2011), as observed in piriform cortex (Poo and Isaacson, 2009; Stettler and Axel, 2009; Stokes and Isaacson, 2010).

The zebrafish brain contains distinct clusters of DA neurons that have been mapped to homologous populations of DA neurons in the mammalian brain (Kaslin and Panula, 2001; Rink and Wullimann, 2001; Ma, 2003; McLean and Fetcho, 2004; Wullimann and Mueller, 2004; Sallinen et al., 2009; Kastenhuber et al., 2010; Tay et al., 2011; Yamamoto et al., 2011). Among these are a population of interneurons in the OB that co-express DA and GABA. These DA neurons may locally modulate input-output of mitral cells (Bundschuh et al., 2012), but do not project to higher brain areas. Additional clusters of DA neurons include a population in the subpallium that is prominent in zebrafish but sparse in mammals and at least one population in the preoptic area. Neurons corresponding to midbrain DA neurons in mammals are thought to be located in the posterior tuberculum in zebrafish, which is part of the diencephalon (Rink and Wullimann, 2001; Wullimann and Mueller, 2004). Dp does not appear to contain dopaminergic somata, but fibers expressing tyrosine hydroxylase (TH), the rate-limiting enzyme of catecholamine synthesis, have been described, suggesting that Dp receives dopaminergic innervation from other brain areas (Castro et al., 2006).

We examined dopaminergic inputs to Dp and effects of DA on synaptic transmission and odor-evoked activity patterns in adult zebrafish. We found that Dp receives dopaminergic projections from multiple groups of DA neurons in the telencephalon and diencephalon. DA decreased inhibitory, but not excitatory, synaptic currents evoked by stimulation of the olfactory tracts, and enhanced responses to odor stimulation. These results indicate that DA can modulate sensory processing in Dp, which may modify sensory perception and plasticity.

## Materials and methods

### Animals, odor delivery, and pharmacology

Experiments were performed in an *ex-vivo* preparation of the nose and brain from adult zebrafish as described (Zhu et al., 2012). Briefly, fish were cold-anesthetized, decapitated, and the ventral forebrain was exposed by removal of the jaws and palate. The preparation was then transferred into a custom chamber (Zhu et al., 2012), continuously perfused with teleost artificial cerebrospinal fluid (ACSF) (Mathieson and Maler, 1988), and warmed up to room temperature. All experimental protocols were approved by the Veterinary Department of the Canton Basel-Stadt (Switzerland). Most experiments were performed in a transgenic line expressing GFP under the control of the vesicular glutamate transporter 2a (vglut2a) promoter, a marker for glutamatergic neurons (Satou et al., 2012). If experiments were performed exclusively in this line, the number of vglut2a-GFP-positive neurons recorded is indicated in the figure legends.

Odors were introduced into a constant stream of medium directed at the ipsilateral naris using a computer-controlled HPLC injection valve (Rheodyne). Odor applications lasted approximately 3 s and were separated in time by 120–180 s to minimize adaptation.

DA was prepared as a stock solution (50 mM, Sigma) together with ascorbic acid (50 mM, Sigma) to prevent oxidation and stored at −20°C. Immediately before application, the stock was thawed and diluted to a final concentration of 50 μM. S-(-)-sulpiride (Sigma) was prepared as a stock solution of 100 mM in DMSO and diluted to a final concentration of 50 μM with 0.05 % DMSO. A previous study showed that ascorbic acid or sulpiride had no detectable effects on neurons in the OB (Bundschuh et al., 2012). The effects of DA were assessed after 5 min of bath application of DA. Drugs were washed out for at least 8 min.

### Electrophysiology and calcium imaging

Recordings were performed using borosilicate pipettes (8–12 MOhms), a Multiclamp 700 B amplifier (Molecular Devices) and Ephus software (Suter et al., 2010). Neurons were targeted by a combination of multiphoton fluorescence and contrast-enhanced transmitted-light optics. Pipettes were filled with an intracellular solution containing 130 mM potassium gluconate, 10 mM sodium gluconate, 10 mM sodium phosphocreatine, 4 mM sodium chloride, 4 mM magnesium-ATP, 0.3 mM sodium-GTP, 10 mM HEPES (pH 7.2, 300 mOsm) and 50 μM Alexa Fluor 594 (Invitrogen). Measurements were not corrected for the liquid junction potential and signals were recorded at 10 kHz. All recordings were performed posterior to the anterior commissure and posterior to the large blood vessel that traverses Dp in medial-lateral direction.

Electrical stimulation of the olfactory tracts was performed using glass pipettes (2–4 MΩ) filled with 1 M NaCl. Stimulus pulses were 0.5 ms in duration and adjusted in amplitude to evoke responses of intermediate magnitude (usually approximately −20 V). To examine the dependence of the response on stimulus strength the pulse amplitude was varied between −1 V and −20 V, in some cases up to −40 V. The olfactory tract of zebrafish consists of many small fiber bundles that are spread out along the medio-lateral extent of the ventral telencephalon. In most cases, the stimulation electrode was placed onto a medial bundle near the OB. Stimulation of lateral bundles was performed in a subset of experiments and gave similar results.

Bolus loading of rhod-2-AM and multiphoton calcium imaging was performed as described (Yaksi and Friedrich, 2006). The multiphoton microscope used has been described in detail before (Zhu et al., 2012). Data were acquired at a rate of 256 ms/frame using scanimage software (Pologruto et al., 2003). Laser intensity was adjusted to minimize photobleaching. Calcium signals (ΔF/F) were calculated as changes in fluorescence intensity (ΔF) relative to a baseline period of 2 s before stimulus onset (F). To quantify calcium signals of individual neurons, regions of interest were outlined manually on time-averaged maps around foci that matched the size and shape of individual somata.

### Immunocytochemistry and retrograde tracing

**Figure 1:**
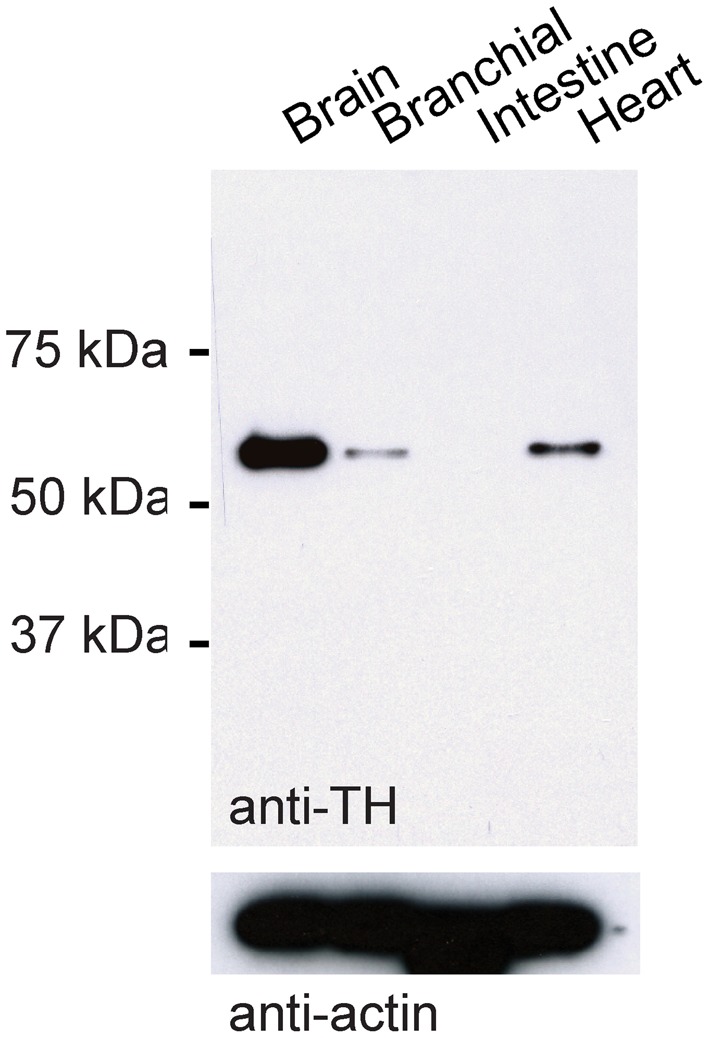
**Western blot control for TH antibody.** Western blot using TH antibody and protein extracts from different tissues of adult zebrafish. Bottom: loading control using antibody against actin.

We used a mouse antibody against tyrosine hydroxylase (TH; 1:200, Millipore MAB318) and various secondary antibodies (1:500, Invitrogen). The monoclonal antibody against TH was raised against TH from PC12 cells and recognizes an epitope on the outside of the regulatory N-terminus. On a Western blot using protein extracts from the adult zebrafish brain, the antibody detected a single band that corresponded to the expected size of the TH protein (59–61 kDa; Figure 1). Moreover, the antibody detected bands of the same size in protein extracts from branchial tissue and from the heart where TH is expected to be expressed (Malvin and Dail, 1986; Parrish et al., 2010), but not in protein extracts from the zebrafish intestine where TH is absent (Olsson et al., 2008). In sections of the OB, the antibody stained a sparse set of neurons at high contrast (not shown). This staining pattern was indistinguishable from the staining pattern obtained previously with a polyclonal antibody raised against zebrafish TH (Bundschuh et al., 2012). These results indicate that the antibody used is specific for TH in zebrafish.

Adult zebrafish brains were fixed in 2% Formal-Fixx (Thermo Scientific, Ref 9990244) in phosphate-buffered saline (PBS) at 4°C for 2 days, cryoprotected in 30% sucrose at 4°C overnight, frozen, and cut on a cryostat (Microm HM560, Leica) into sections of 14 μM thickness. Immunocytcochemical staining of cryosections was performed with a Ventana Discovery XT automated system (Ventana Medical Systems) following the manufacturer's protocol with proprietary reagents. Slides were initially rinsed with reaction buffer (Ventana). The monoclonal antibody against TH was used at 1:200 and incubated for 60 min at 37°C. Slides were rinsed again with Ventana reaction buffer before the fluorescent secondary antibody was applied at 1:500 dilution and incubated for 32 min at 37°C. Slides were rinsed again and mounted using Prolong anti-fade gold reagent (Invitrogen).

For Western blot analysis, four adult wild-type zebrafish were anesthetized using ethyl 3-aminobenzoate methanesulfonate (MS-222; 0.01% w/v) and euthanized. Organs (brain, branchial tissue, intestine, and heart) were removed in ice-cold phosphate-buffered saline (PBS). Ice-cold RIPA lysis buffer including phenylmethanesulfonyl fluoride (PMSF) and a protease inhibitor cocktail (Roche) was added and tissue was homogenized with a pestle on ice for 1 min. The tissue was left on ice for 4 min and homogenization was repeated for 1 more min. The lysate was incubated for approximately 1 h with shaking at 4°C. Lysates were centrifuged at 4°C, 17,000 g, for 8 min and the pellet was discarded. Total protein content was quantified using the Bradford quickstart reagent kit (Bio-Rad). Approximately 10 μg per lysate was used. After addition of loading buffer containing dithiothreitol (DTT), samples were heated to 70°C for 7 min, spun down, and loaded onto 10% polyacrylamide gels. Proteins were then transferred onto nylon membranes at 200 mA for 1 h in the cold (approximately 4°C), blocked in 5% milk in Tris-buffered saline and Tween 20 (TBST), and incubated overnight at 4°C with the anti-TH antibody (1:1000). The secondary horseradish peroxidase-conjugated antibody against species-specific IgGs was used at 1:10,000 dilution. The chemiluminescent signal was detected using Immobilon reagent (Millipore).

Retrograde neuronal tracers, biotinylated dextran amine (Dextran-biotin, 10,000 MW, Invitrogen) and dextran tetramethylrhodamine (Dextran-rhodamine, 3000 MW, Invitrogen), were mixed and crystallized onto the tip of an insect pin (Fine Science Tools, tip diameter 0.25 mm). The pin was stabbed into the ipsilateral Dp in the *ex-vivo* preparation and removed again within a few seconds. The preparation was then transferred into a perfusion chamber at room temperature, superfused with ACSF, and inspected by multiphoton microscopy to monitor the spread of the tracer. Within less than 1 h, dextran tetramethylrhodamine was detected in mitral cell somata in the OB, which are further away than most clusters of DA neurons. At least 3 h after dye application the brain was fixed for immunocytochemistry as described above. Extrinsic sources of DA projections to Dp were identified by combining TH and streptavidin (Alexa Fluor 568, streptadvidin conjugate, Invitrogen, S11226) immunocytochemistry. Confocal images were acquired with a Zeiss LSM 510 confocal laser scanning microscope.

### Data analysis

Data analysis was performed using custom routines written in Matlab. Synaptic currents evoked by electrical stimulation were averaged over 30–40 trials. The synaptic latency was estimated as the time between stimulus onset and the first inflection of the current trace (see Figure 3A). The inflection point was usually sharply defined within a time window of < 2 ms and determined manually by inspection of each trace. The amplitudes of averaged EPSCs and IPSCs are the mean currents relative to pre-stimulus baseline within an 18 ms time window following the stimulus. The paired-pulse ratio was determined by dividing the amplitude of the second response by the amplitude of the first response. Input resistance was calculated by measuring peak and steady-state current responses to −30 mV voltage steps (100 ms) and dividing the voltage step by the current response (see Figure 4A). Amplitudes of time-averaged calcium signals were quantified as the mean ΔF/F during a 3 s time window following stimulus onset. Other time windows gave similar results. Somatic responses were analyzed quantitatively only for those neurons that responded to at least one stimulus condition. Results are reported as mean ± standard deviation (SD) unless indicated otherwise. Statistical significance was assessed using a non-parametric Wilcoxon rank-sum test for unpaired samples and a non-parametric sign-rank test for paired samples.

## Results

### Dopaminergic input to Dp

A previous study reported a sparse population of axons in Dp of adult zebrafish that expressed TH, a marker for catecholaminergic neurons, and appeared to originate in the subpallium (Castro et al., 2006). Other studies, however, did not report TH-positive axons in Dp of adult zebrafish (Kaslin and Panula, 2001; Ma, 2003), and single-neuron genetic tracing failed to identify projections of DA neurons to the presumed location of Dp in zebrafish larvae (Kastenhuber et al., 2010; Tay et al., 2011). We therefore used fluorescence immunocytochemistry in combination with neuronal tracing to clarify whether Dp receives dopaminergic input in the adult zebrafish brain.

**Figure 2:**
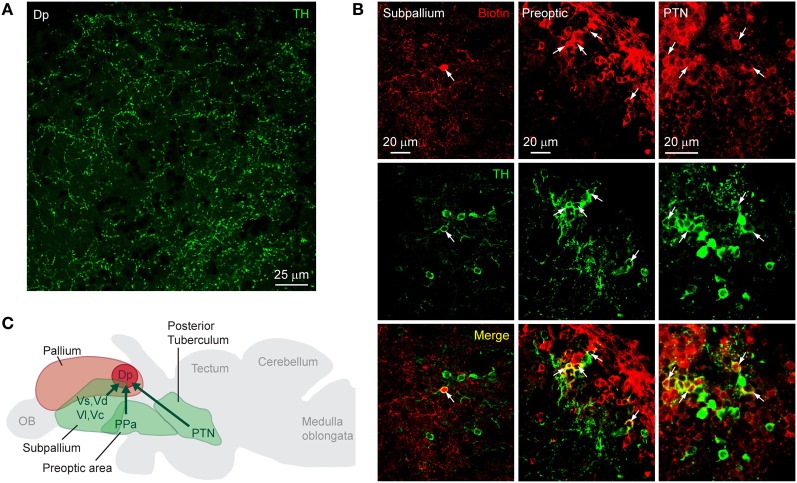
**Dopaminergic input to Dp. (A)** Expression of TH in an optical section through Dp (confocal image). **(B)** Distribution of tracer (biotin-dextran; red) after injection into Dp and immunocytochemical staining against TH (green) in three brain areas [subpallium, preoptic area, and a diencephalic area that is likely to be the PTN (posterior tuberal nucleus); confocal images]. Neurons double-positive for the tracer and TH (yellow) were found in all three brain areas. **(C)** Summary of results. TH-positive neurons that project to Dp were found in the subpallium, the preoptic area, and an area in the midbrain that appears to correspond to a nucleus in the posterior tuberculum (PTN).

On cryosections of the adult brain (14 μm), an antibody against TH stained characteristic clusters of neurons in the telencephalon and diencephalon, closely matching expression patterns reported previously (Kaslin and Panula, 2001; Rink and Wullimann, 2001; Ma, 2003; Wullimann and Mueller, 2004). In Dp, no fluorescent somata were found but a sparse meshwork of varicose fibers was clearly present (Figure 2A). To determine the origin of these fibers we labeled neurons projecting to Dp from other brain areas by focal applications of biotin-dextran and rhodamine-dextran into Dp (*n* = 4 fish). Within Dp, neuronal somata were densely labeled within a diameter of 300 μm or less, confirming that dye applications were local (not shown). Subsequently, DA neurons were identified by immunocytochemistry against TH. We focused on three well-described populations of DA neurons: (1) subpallial DA neurons. This group is located in or near the central, lateral, dorsal, and supracommissural nuclei of the ventral telencephalon (Vc, Vl, Vd, and Vs, respectively) (Kaslin and Panula, 2001; Rink and Wullimann, 2001; Ma, 2003) and has been reported to send fibers toward Dp (Castro et al., 2006). (2) DA neurons in the preoptic area, particularly in or near the anterior parvocellular preoptic nucleus (PPa). These neurons are located ventromedially to Dp, express high levels of TH, and project in various directions (Ma, 2003). (3) DA neurons in the posterior tuberculum, which contains six or more distinct groups of DA neurons. At least two of these groups are likely to project to striatal areas in the subpallium, and another subgroup has been suggested to project to pallial targets (Rink and Wullimann, 2001; Wullimann and Rink, 2002).

Neuronal somata double-labeled by the tracer and the antibody against TH were found in the subpallium, the preoptic area, and in a diencephalic area just dorsal to the hypothalamus that probably corresponds to the posterior tuberal nucleus (PTN) of the posterior tuberculum (Figure 2B). In each brain area, additional neurons were detected that stained only for the tracer or only for TH (Table 1). Dp therefore appears to receive dopaminergic and non-dopaminergic input from all three brain areas examined (Figure 2C).

**Table 1:** **Tracing of neurons that project to Dp and express TH**.

	**Preoptic region (ipsilateral)**	**Preoptic region (contralateral)**	**Subpallium (ipsilateral)**	**Diencephalon (ipsilateral)**
Tracer-positive	97	93	23	50
TH-positive	81	102	75	23
Double-positive	16	12	8	8

The top rows report the numbers of neuronal somata labeled by tracer injection into Dp and the number of TH-positive neuronal somata in four different brain regions (n = 4 fish). The last row shows the number of double-positive neurons (included in the total counts above). Tracer was applied into Dp on one side of the animal (ipsilateral). Numbers do not reflect the total numbers of neurons in each brain area because only slides containing double-positive neurons were counted. No double-positive neurons were detected in the subpallium or the diencephalon contralateral to the tracer injection site.

### Synaptic input to Dp neurons

To examine the dopaminergic modulation of synaptic responses and activity patterns in Dp we first analyzed synaptic currents in an *ex-vivo* preparation of the intact zebrafish brain and nose (Zhu et al., 2012). Input to Dp was evoked by electrical stimulation of the olfactory tract, which contains axons of mitral cells projecting to higher brain areas and axons of telencephalic neurons projecting back toward the OB. Because the olfactory tract in zebrafish consists of multiple segregated axon fascicles, stimulation at a given site most likely activates only a subset of mitral cell axons. Most recordings were performed in voltage clamp from Dp neurons expressing GFP under the control of the vglut2a promoter, a marker of glutamatergic neurons (Satou et al., 2012).

**Figure 3:**
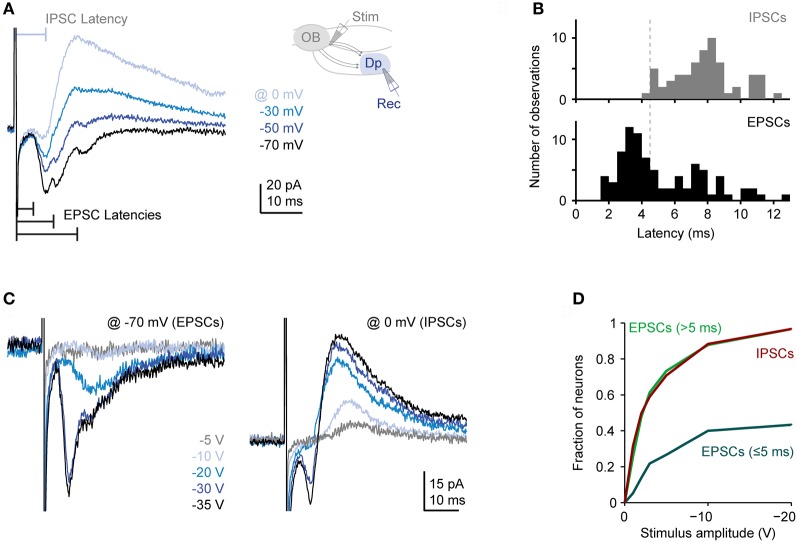
**Synaptic currents evoked in Dp neurons by electrical stimulation of the olfactory tract. (A)** Family of currents recorded in whole-cell voltage clamp at different holding potentials (electrical stimulation of medial olfactory tract; stimulus amplitude: −30 V). Inset shows stimulation and recording configuration. Inward currents recorded at −70 mV (EPSCs) frequently had multiple components with distinct peaks. Outward currents recorded at 0 mV (IPSCs) did not show distinct peaks and lacked a short-latency component. **(B)** Distributions of EPSC and IPSC latencies evoked by electrical stimulation of the medial olfactory tract (*n* = 83 neurons in 38 fish; the majority was vglut2a-GFP-positive). If a compound current response had multiple distinct components, only the shortest latency was counted. Stimulation amplitude was −20 to −30 V. **(C)** Examples of EPSCs and IPSCs evoked by stimuli of different intensities. Note recruitment of additional response components with increasing stimulus amplitude. **(D)** Fraction of neurons showing a short-latency (presumably monosynaptic) EPSC, at least one long-latency (presumably polysynaptic) EPSC, and an IPSC as a function of stimulus amplitude (*n* = 30 neurons). If a compound current response had multiple distinct components, all components were considered.

Single voltage pulses to the olfactory tract evoked a complex sequence of inward currents recorded at −70 mV [excitatory postsynaptic currents (EPSCs)] and outward currents recorded at 0 mV or −10 mV [inhibitory postsynaptic currents (IPSCs)], consistent with previous observations (Blumhagen et al., 2011). Inward currents often consisted of multiple components whose onsets and peaks were clearly separated in time (Figure 3A). The distribution of latencies (*n* = 83 neurons in 38 fish) had an early peak and a long tail (Figure 3B). The early peak represents a short latency component (< 5 ms) that was observed in a subset of responses while the tail represents delayed excitatory input (latencies > 5 ms) that was present in most responses. These components most likely reflect monosynaptic input from mitral cells (and possibly other neurons with axons in the olfactory tract) and polysynaptic inputs from the Dp network, respectively (Poo and Isaacson, 2011). Outward currents, in contrast, rarely had distinct components, were more extended in time, and lacked a short-latency component (Figures 3A,B; *n* = 83 in 38 fish). Inhibitory synaptic input is therefore predominantly or exclusively polysynaptic, presumably because inhibitory interneurons are recruited by glutamatergic neurons. These results are consistent with recent observations in the olfactory cortex of rodents (Poo and Isaacson, 2011).

We next determined the dependence of synaptic currents on stimulus intensity. On average, the magnitude of all current components increased with stimulus intensity but short- and long-latency components were recruited differentially (Figures 3C,D). As stimulus intensity was increased, the probability of observing a short-latency inward current increased initially but then saturated around 40%. The probability of observing long-latency inward or outward currents, in contrast, increased more rapidly with stimulus intensity and eventually approached 100% (*n* = 30 neurons; Figure 3D). Hence, polysynaptic excitatory and inhibitory input from the Dp network is dense and occurs frequently even when a Dp neuron is not activated by electrical stimulation of direct mitral cell input. This observation is consistent with the broad tuning of intra-cortical excitation and inhibition to odors in piriform cortex (Poo and Isaacson, 2009, 2011; Franks et al., 2011).

### Modulation of synaptic transmission by DA

**Figure 4:**
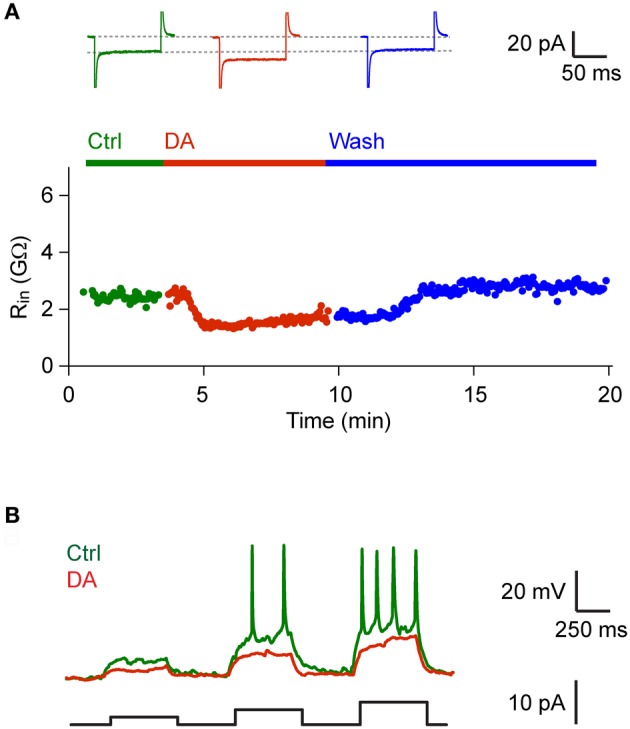
**DA decreased input resistance in a subset of Dp neurons. (A)** Input resistance as a function of time before, during and after bath-application of DA (50 μM) in a Dp neuron (vglut2a-GFP-positive). Top: currents measured in voltage clamp in response to voltage steps (−30 mV) before, during and after exposure to DA. Similar effects of DA on input resistance were observed in a minority of Dp neurons (6/44 neurons; all vglut2a-GFP-positive). **(B)** Responses to current steps of increasing amplitude recorded in current clamp before and during exposure to DA in the same neuron.

We next examined effects of DA on neurons and synaptic currents in Dp. Bath-application of DA (50 μM) had no significant effect on the mean holding current (control: −7.04 ± 6.43, DA: −7.00 ± 5.00; holding potential: −70 mV; *n* = 44 neurons in 19 fish, 42/44 neurons vglut2a-GFP-positive) and input resistance (control: 2.10 ± 1.58 GΩ, DA: 2.05 ± 1.42 GΩ). However, in 6 out of 44 neurons (all vglut2a-GFP-positive) we noted a decrease in input resistance that resulted in an increased rheobase (Figure 4). This effect was reversible after wash-out (*n* = 4 neurons in 4 fish; Figure 4A) or after wash-in of an antagonist of D1-like DA receptors (SCH 23390, 50 μM; *n* = 2 neurons in 2 fish; not shown).

**Figure 5:**
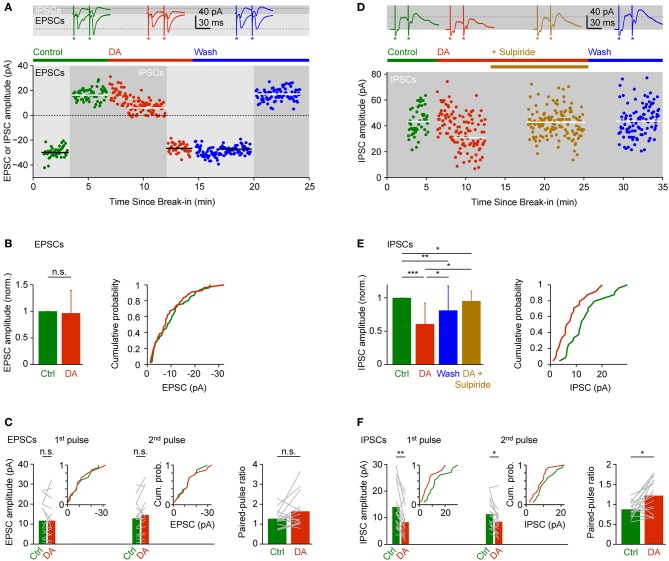
**Effects of DA on EPSCs and IPSCs evoked by electrical stimulation of the olfactory tract. (A)** EPSCs and IPSCs in a Dp neuron evoked by electrical stimulation before, during and after bath-application of DA (50 μM). EPSCs were recorded at a holding potential of −70 mV during the periods indicated by light gray shading; IPSCs were recorded at 0 mV during periods indicated by dark gray shading. Horizontal lines indicate mean EPSC or IPSC amplitudes. Top: examples of EPSCs and IPSCs evoked by pairs of stimuli before (green), during (red) and after (blue) exposure to DA. Asterisks indicate stimulus artifacts (30 ms inter-stimulus interval). Dashed lines indicate amplitude of EPSC and IPSC evoked by the first pulse before DA. In the scatter plot, current amplitude was quantified in response to the first pulse only. **(B)** Mean normalized EPSC amplitude (± SD) before and during application of DA (left; *n* = 55 neurons in 28 fish, 53/55 neurons vglut2a-GFP-positive) and cumulative probability distribution of EPSC amplitudes before (green) and during (red) exposure to DA. If currents were evoked by pairs of pulses, only the response amplitude evoked by the first pulse was quantified. **(C)** Mean EPSC amplitude evoked by the first and second pulse (left) and mean paired-pulse ratio (right) before and during exposure to DA (*n* = 16 neurons in 9 fish, 15/16 neurons vglut2a-GFP-positive). Gray lines show changes in individual neurons. Insets show cumulative probability distributions of EPSC amplitudes. **(D)** Effects of DA and sulpiride on IPSCs in a different Dp neuron. In the scatter plot, current amplitude was quantified in response to the first pulse only. **(E)** Mean normalized IPSC amplitude (± SD; left) and cumulative probability distribution of IPSC amplitudes before (green) and during (red) exposure to DA. Effect of DA and washout was tested in *n* = 17 neurons from 10 fish (all vglut2a-GFP-positive). Effect of DA and sulpiride was tested in an additional *n* = 4 neurons from 4 fish (all vglut2a-GFP-positive). If currents were evoked by pairs of pulses, only the response amplitude evoked by the first pulse was quantified. **(F)** Mean IPSC amplitude evoked by the first and second pulse (left) and mean paired-pulse ratio (right) before and during exposure to DA (*n* = 16 neurons in 9 fish, 15/16 vglut2a-GFP-positive). Insets show cumulative probability distributions of IPSC amplitudes. ^*^P < 0.05; ^**^P < 0.01; ^***^P < 0.001; n.s., not significant.

DA had no obvious effects on EPSCs evoked by olfactory tract stimulation. The mean amplitude of inward currents recorded at −70 mV was not significantly different from control (96 ± 43% of control), with considerable variability between experiments (Figures 5A,B; *n* = 55 neurons in 28 fish). The amplitude ratio of currents evoked by pairs of pulses (30 ms interval) was slightly larger in the presence of DA (Figure 5C; control: 1.25 ± 0.43; DA: 1.60 ± 0.81; *n* = 16 neurons in 9 fish) but this effect was not statistically significant.

IPSCs recorded at 0 mV or −10 mV, in contrast, were significantly reduced to 61 ± 31% of control in the presence of DA (Figures 5A,D,E; *n* = 24 neurons in 24 fish). This effect was reversible after wash-out (Figures 5A,D,E; 81 ± 37% of control; *n* = 17 neurons in 10 fish) and blocked by addition of sulpiride (50 μM), an antagonist of D2-like DA receptors (Figures 5D,E; 95 ± 15% of control; *n* = 4 neurons in 4 fish). The paired-pulse ratio of IPSCs increased significantly from 0.88 ± 0.20 under control conditions to 1.22 ± 0.42 in the presence of DA (Figure 5F; *n* = 16 neurons in 9 fish). Hence, DA selectively reduced inhibitory synaptic transmission in Dp, which may involve a presynaptic mechanism and D2-like DA receptors. These results were essentially unchanged when only vglut2a-GFP-positive neurons were included in the analysis.

**Figure 6:**
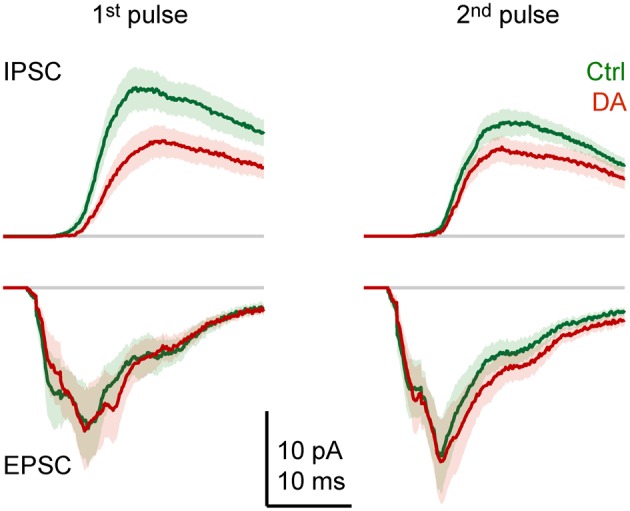
**Effects of DA on the time course of synaptic currents.** EPSCs and IPSCs evoked by paired-pulse stimulation of the olfactory tract (inter-stimulus interval: 30 ms) before (green) and during (red) bath-application of DA (50 μM), averaged over Dp neurons (*n* = 16 in 9 fish). Stimulus artifacts were removed. Lines show mean current; shading shows standard error.

Effects of DA on the dynamics of the compound synaptic response were examined by comparing the time course of averaged synaptic currents evoked by pairs of pulses (Figure 6). As observed in currents from individual neurons (Figure 3), the average inward current evoked by the first pulse consisted of multiple components: a short-latency (monosynaptic) component, an intermediate component with a clear peak, and at least one late component. The response to the second pulse was similar, but the amplitude of the intermediate component was increased. Outward currents lacked a short-latency component, were more prolonged, and the response to the second pulse was reduced in amplitude. The most obvious effect of DA was a general reduction of outward currents, particularly in response to the first pulse. In addition, DA had subtle effects on the time course of inward currents. Most notably, the short-latency component evoked by the first pulse was slightly reduced while the late component was enhanced. In response to the second pulse, DA slightly prolonged the late components.

### Effects of DA on population activity patterns

**Figure 7:**
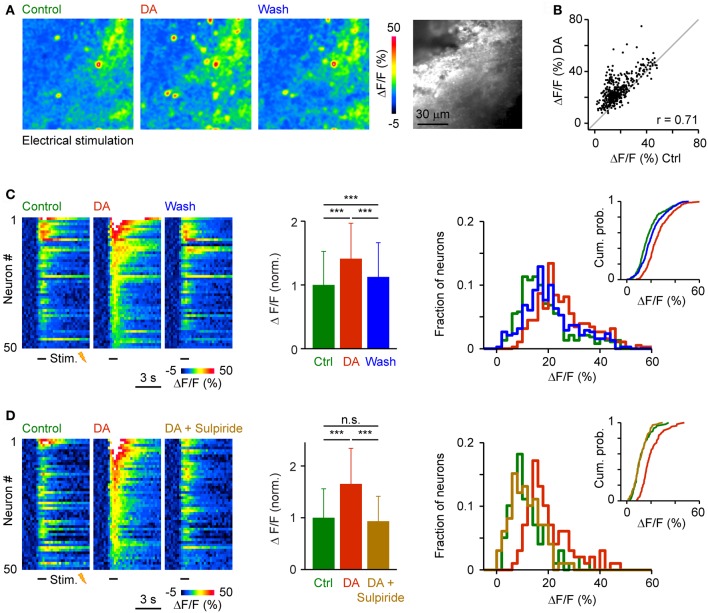
**Effects of DA on population activity patterns in Dp evoked by electrical stimulation of the olfactory tract. (A)** Time-averaged spatial patterns of calcium signals evoked by olfactory tract stimulation (20 Hz for 1 s) before, during and after bath-application of DA, measured by multiphoton microscopy. **(B)** Calcium signals of all cells (*n* = 380) before (*x*-axis) and during (*y*-axis) exposure to DA. Gray: line with slope 1. r, Pearson correlation coefficient. **(C)** Left: Calcium signals of 50 randomly selected neurons as a function of time, sorted by magnitude in the presence of DA. Center: Mean amplitude of calcium signals before, during and after exposure to DA (*n* = 380 from 3 fish; normalized). Right: Distribution of response amplitudes before, during and after DA exposure. Inset shows cumulative probability distribution. **(D)** Calcium signals of 50 randomly selected neurons (left), mean calcium signals of all neurons (*n* = 99 from 2 fish; center), and amplitude distributions (right) before DA, during the presence of DA, and after addition of sulpiride (50 μM). ^***^P < 0.001; n.s., not significant.

To examine effects of DA on population activity patterns we injected the red-fluorescent calcium indicator, rhod-2-AM, into Dp and measured somatic calcium signals by multiphoton microscopy (Yaksi and Friedrich, 2006; Yaksi et al., 2009; Blumhagen et al., 2011). We first applied trains of electrical stimuli (1 s, 20 Hz, 3 repetitions) to the olfactory tract to bypass effects of DA on the OB (Bundschuh et al., 2012). Responses were averaged over repetitions and quantified in regions of interest matching the size and shape of neuronal somata. After wash-in of DA, many responses were increased, a few responses were unchanged or reduced, and some neurons were newly recruited into the pattern. Nevertheless, the overall activity pattern across the population remained similar (Figures 7A,B). The Pearson correlation between somatic calcium signals during exposure to DA and calcium signals evoked before or after DA application was 0.71 and 0.75, respectively. The Pearson correlation between calcium signals before and after DA application was 0.82. Hence, response patterns across the population of Dp neurons were reorganized modestly in the presence of DA.

**Figure 8:**
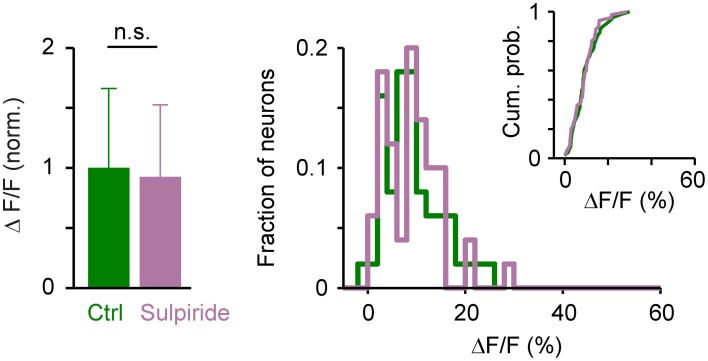
**Effects of sulpiride on population activity patterns in Dp evoked by electrical stimulation of the olfactory tract.** Left: Mean amplitude of calcium signals before and during exposure to sulpiride (*n* = 50 neurons from 2 fish; normalized). Right: distributions of calcium signal amplitudes; inset shows cumulative probability distribution. No statistically significant differences were detected between means or distributions.

The amplitude of somatic calcium signals was, on average, significantly increased in the presence of DA to 141 ± 56% of control (*n* = 380 cells) and the distribution of response amplitudes was shifted toward higher values (Figures 7C,D). These effects were reversed after wash-out of DA (113 ± 54% of control) or by addition of sulpiride (DA: 165 ± 69% of control; DA + sulpiride: 93 ± 48% of control; *n* = 99 cells in 2 fish; Figure 7D). Sulpiride alone had no detectable effect on response amplitudes and their distribution (Figure 8). The main effect of DA was therefore an enhancement of responses within a substantial subpopulation of Dp neurons.

**Figure 9:**
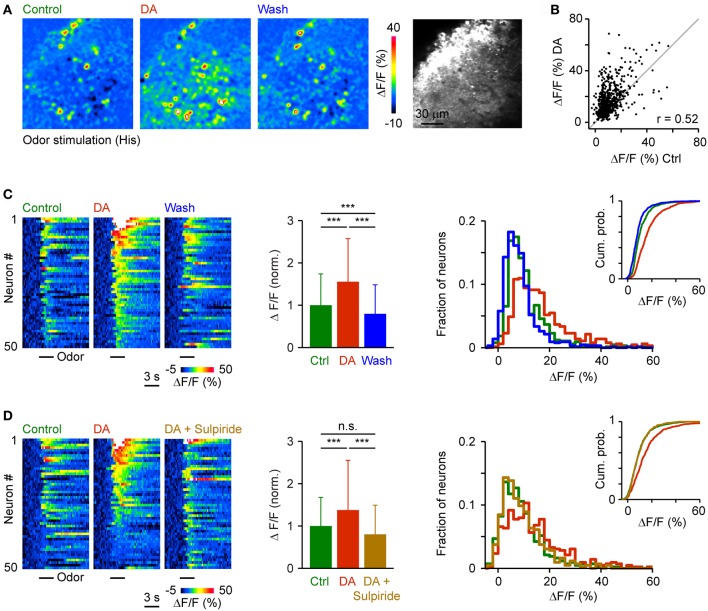
**Effects of DA on odor-evoked population activity patterns in Dp. (A)** Time-averaged spatial patterns of calcium signals evoked by odor stimulation (His, 10 μM) before, during and after bath-application of DA, measured by multiphoton microscopy. **(B)** Calcium signals of all cells (*n* = 658) before (*x*-axis) and during (*y*-axis) exposure to DA. Gray: line with slope 1. r, Pearson correlation coefficient. **(C)** Left: Calcium signals of 50 randomly selected neurons as a function of time, sorted by magnitude in the presence of DA. Center: Mean amplitude of calcium signals before, during and after exposure to DA (*n* = 658 neurons from 20 fish; normalized). Right: Distribution of response amplitudes before, during and after DA exposure. Inset shows cumulative probability distribution. **(D)** Calcium signals of 50 randomly selected neurons (left) and mean calcium signals of all neurons (*n* = 278 neurons from 8 fish; center) and amplitude distributions (right) before DA, during the presence of DA, and after addition of sulpiride (50 μM). ^***^P < 0.001; n.s., not significant.

We then analyzed effects of DA on activity patterns evoked by an amino acid odor at an intermediate concentration (His, 10 μM; 3 repetitions averaged). DA reversibly increased responses of many neurons while some responses were unchanged or reduced (Figures 9A,B). As observed in response to electrical stimulation, DA changed the pattern of activity across the population of Dp neurons only modestly. The Pearson correlation between somatic calcium signals during exposure to DA and calcium signals evoked before or after DA application was 0.52 and 0.55, respectively, while the Pearson correlation between calcium signals before and after DA application was 0.65. Activity patterns in the presence of DA were therefore not identical but still related to response patterns under control conditions.

Response amplitudes in the presence of DA were shifted toward higher values and, on average, significantly increased to 155 ± 102% of control (658 neurons in 20 fish). After wash-out, the distribution of response amplitudes shifted back toward lower values and the mean response amplitude returned to 80 ± 69% of control (Figure 9C). In an additional set of experiments (*n* = 278 neurons in 8 fish), the increase in response amplitude and the shift in the amplitude distribution in the presence of DA (138 ± 118% of control) was reversed by addition of sulpiride (Figure 9D; 80 ± 69% of control). DA therefore enhanced and partially reorganized odor-evoked activity patterns in Dp, consistent with its effect on activity patterns evoked by electrical stimulation.

**Figure 10:**
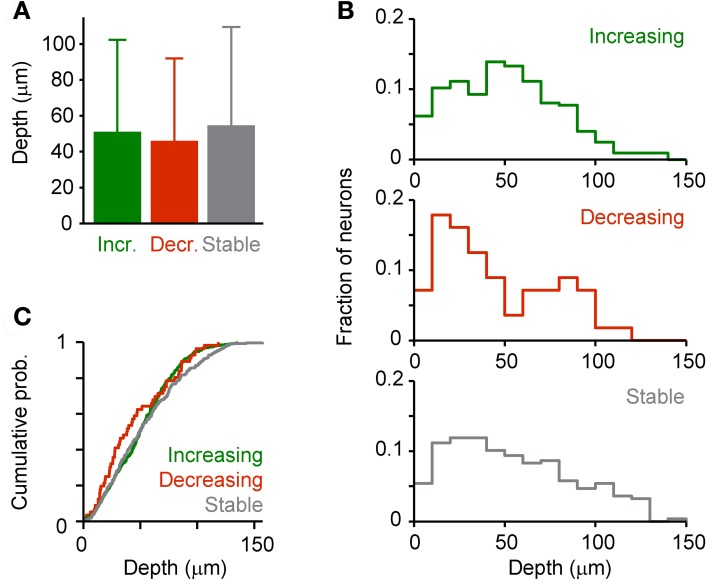
**Depth-dependence of DA effects.** Dp neurons were assigned to one of three categories (“Increasing,” “Decreasing,” “Stable”) based on the effect of DA on the response evoked by an amino acid odor (His, 10 μM; see text). **(A)** Mean depth of neurons in each category. **(B)** Depth distribution of each category. **(C)** Cumulative probability distributions. No statistically significant differences were detected between means or distributions.

In order to examine whether effects of DA on response amplitudes vary with the depth below the surface we grouped neurons that responded to odor stimulation (His; *n* = 658) into three categories based on the effect of DA: (1) Neurons, whose response increased by at least 50% (“Increasing”; *n* = 324), (2) neurons whose response decreased by at least 50% (“Decreasing”; *n* = 56), and (3) the remaining neurons (“Stable”; *n* = 278). We found no category-dependent difference in the mean depth of neurons or between their depth distributions (Figure 10). However, this result does not exclude the possibility that DA modulates specific subsets of neurons because layering of neuron types in Dp is not as pronounced as in mammalian cortices.

## Discussion

DA is an important neuromodulator that can change the strength or dynamics of synaptic transmission and modulate the induction or maintenance of synaptic plasticity in a wide range of cortical and subcortical brain areas (Otmakhova and Lisman, 1996, 1998; Gurden et al., 2000; Schultz, 2002; Pawlak et al., 2010). Despite the widespread importance of the dopaminergic system, however, its influence on olfactory cortex is still unclear. We therefore began to address this issue in zebrafish, which combines advantages of a genetic model organism with a small, optically accessible brain.

### Dopaminergic input to Dp

Although the location and development of DA neurons in zebrafish has been studied extensively, their projections to pallial brain areas have not been analyzed in detail. In particular, it remained unclear whether DA neurons project to Dp in adult zebrafish. We found neurons that projected to Dp and expressed TH in three brain areas: parts of the subpallium (around Vc, Vl, Vd, and Vs), the preoptic region, and a diencephalic area that appears to be the PTN of the posterior tuberculum. TH-expressing neurons in these brain areas are dopaminergic because other catecholamines are not expressed anterior to the hindbrain (Kaslin and Panula, 2001; Yamamoto et al., 2011). Our results do not allow for a quantitative analysis of the fraction of TH-expressing neurons that project to Dp because the tracer was probably delivered only to a subset of these neurons, and because we did not reconstruct complete clusters of TH-expressing neurons. Nevertheless, the results show that Dp receives input from DA neurons in at least three distinct brain areas. These projections may arise late in development because axonal projections to presumed pallial target areas have not been observed by genetic tracing of individual DA neurons in early zebrafish larvae (Kastenhuber et al., 2010; Tay et al., 2011). These results indicate that the function of neuronal circuits in Dp is likely to be influenced by DA neurons, consistent with our physiological results.

While DA neurons in the subpallium and in the preoptic region of zebrafish have direct counterparts in the mammalian brain (Bjorklund and Dunnett, 2007), the mammalian equivalents of DA neurons in the posterior tuberculum are still debated. Based on tracing results and comparative studies it has been suggested that ascending DA neurons of the posterior tuberculum correspond to midbrain DA neurons in mammals and, thus, represent the mesostriatal, mesolimbic and possibly the mesocortical systems in zebrafish (Rink and Wullimann, 2001, 2002; Wullimann and Mueller, 2004). We found a small population of dopaminergic neurons in the diencephalon that projected to Dp but appeared to be different from DA neurons projecting to striatal targets. We assume that these neurons are located in the PTN of the posterior tuberculum, which would provide further support for a correspondence between DA neurons in the posterior tuberculum of teleosts and the mammalian midbrain. However, the precise identity of Dp-projecting DA neurons in the diencephalon should be examined in more detail in a separate study. The finding that multiple dopaminergic cell clusters project to a pallial target area may suggest that DA is involved in multiple functions (Schultz, 2002; Redgrave and Gurney, 2006).

### Dopaminergic modulation of synaptic transmission in Dp

As observed in piriform cortex (Poo and Isaacson, 2009, 2011), electrical stimulation of the olfactory tract evoked a complex sequence of EPSCs in Dp neurons that included a distinct short-latency component and additional components with longer latencies. These components have been attributed to direct monosynaptic input from mitral cells and to delayed polysynaptic input from higher-order neurons, respectively (Poo and Isaacson, 2011). In Dp, the early component may also include synaptic input from antidromically stimulated glutamatergic neurons. Compound IPSCs in Dp lacked a short-latency component, consistent with IPSCs in piriform cortex neurons (Poo and Isaacson, 2011), and had a slower time course. Compound IPSCs may therefore be composed of multiple polysynaptic inputs with some temporal jitter. Long-latency excitatory and inhibitory input was more often recruited by electrical stimulation than short-latency input, suggesting that Dp neurons receive substantial input from other neurons within Dp that is broadly tuned. This finding is consistent with the broad tuning of intra-cortical inputs to odors in piriform cortex neurons (Poo and Isaacson, 2009, 2011) which may arise from distributed connectivity between higher-order neurons (Johnson et al., 2000; Franks et al., 2011). Together, these results show functional similarities between Dp and olfactory cortex, particularly piriform cortex.

The few studies that have examined physiological actions of DA in olfactory cortex reported disparate effects including a reduction of spontaneous firing *in vivo* (Legge et al., 1966), enhanced spontaneous firing of inhibitory neurons *in vitro* (Gellman and Aghajanian, 1993), and dose-dependent changes of evoked field potentials *in vitro* (Collins et al., 1985). We found that bath-application of DA consistently reduced IPSCs in principal neurons of Dp. This effect is unlikely to be caused by actions of DA in the OB (Bundschuh et al., 2012) because it was observed also when the OB was bypassed by stimulating the olfactory tract. Because the reduction in IPSC amplitude was observed already at the earliest time points, DA most likely affected inhibitory synaptic transmission within Dp. It cannot be ruled out that DA also affected inhibitory synaptic inputs to Dp neurons more indirectly by effects in other brain regions. However, such effects, if they exist, would be expected to contribute to later phases of the IPSC because of additional synaptic and conduction delays. The decrease in IPSC amplitude may be due to reduced transmitter release from inhibitory interneurons because it was accompanied by an increased paired-pulse ratio. However, other mechanisms could also account for this effect since IPSCs were polysynaptic.

DA did not change the amplitude of compound EPSCs but had subtle effects on their time course. It is therefore possible that DA also modulated excitatory synaptic transmission in Dp. Alternatively, effects on the time course of compound EPSCs may be an indirect consequence of reduced inhibition. In particular, the enhancement of the late, polysynaptic components could be due to increased activity of excitatory Dp neurons in the presence of DA.

Since DA reduced compound IPSCs but not EPSCs, the overall effect on principal neurons was a disinhibition. Consistent with this finding, DA significantly increased responses to olfactory tract stimulation or odor stimulation in many Dp neurons. An antagonist of D2-like DA receptors blocked the reduction of IPSCs and also abolished the increased response to electrical stimulation or odors. DA therefore enhances responses of Dp neurons to sensory input, most likely by disinhibiting principal neurons via a D2-dependent mechanism. The effect of DA on response amplitudes in Dp was pronounced, consistent with the finding that inhibition is strong during odor responses in Dp (Yaksi et al., 2009) and mammalian piriform cortex (Poo and Isaacson, 2009, 2011). In a subset of Dp neurons, DA also decreased input resistance, which was reversed by an inhibitor of D1-like receptors. Hence, DA is likely to have additional effects that may be further explored in future studies.

### Possible functional consequences of dopaminergic modulation

The modulation of synaptic transmission and activity patterns by DA may have multiple consequences for olfactory processing. The enhancement of sensory responses is likely to increase the impact of Dp output on its target areas, which could directly enhance the saliency of odor representations and affect sensory perception.

In cortical brain areas including piriform cortex, disinhibition has been suggested as a mechanism by which cholinergic inputs control the induction of synaptic plasticity (Kanter and Haberly, 1993; Hasselmo and Barkai, 1995; Patil et al., 1998; Patil and Hasselmo, 1999; Hasselmo, 2006; Letzkus et al., 2011). Both acetylcholine and DA convey information about important external events and have been proposed to act as global teaching signals during learning (Schultz, 2002; Hasselmo, 2006; Redgrave and Gurney, 2006). Models of olfactory cortex suggest that such signals are important for memory formation and recall (Hasselmo et al., 1990; Hasselmo, 1993; Wilson and Sullivan, 2011). Future studies may thus explore whether DA modulates synaptic plasticity in Dp and plays a role in some forms of olfactory learning. Moreover, it may be explored whether DA plays additional roles (Redgrave and Gurney, 2006), and whether DA input from different brain regions mediates different functions. Zebrafish offer the opportunity to explore these questions using opto- and pharmacogenetic approaches (Friedrich et al., 2010; del Bene and Wyart, 2012).

### Conflict of interest statement

The authors declare that the research was conducted in the absence of any commercial or financial relationships that could be construed as a potential conflict of interest.
